# Multifaceted Effects of a Multiple Nitric Oxide Photoreleaser and its Photoproducts on Amyloid‐β Aggregation

**DOI:** 10.1002/cbic.202500667

**Published:** 2025-11-24

**Authors:** Francesca Laneri, Cristina Parisi, Salvatore Sortino

**Affiliations:** ^1^ PhotoChemLab Department of Drug and Health Sciences University of Catania I‐95125 Catania Italy

**Keywords:** Alzheimer, amyloid, light, nitric oxide, organofluoride

## Abstract

Aberrant aggregation of β‐amyloid (Aβ) peptides into insoluble fibrils is recognized as one of the hallmarks of Alzheimer's disease (AD). Among the post‐translational modifications influencing Aβ behavior, nitration and nitrosation by nitric oxide (NO) derivatives play a key role, though their impact on aggregation and toxicity remains unclear. This contribution explores the effects on Aβ aggregation induced by an NO photodonor (NOPD) releasing two NO molecules via a stepwise mechanism under the control of visible blue light. Significant reduction of protein aggregation is observed when a considerable amount NO (≈120 µM) is photoreleased during the early stages of the protein aggregation. In contrast, long‐term release of a similar NO concentration is ineffective. On the other hand, proaggregative effect is induced by the NOPD kept in the dark. The stable photoproducts formed after the release of the first and the second molecule of NO also show a protein aggregation inhibitory effect both individually and in combination. Dynamic simulation studies are also reported to shed light on the binding of NOPD and its photoproducts with key Aβ_1–40_ residues.

## Graphical abstract

A blue light‐activatable NO photodonor sequentially releases two NO molecules accompanied by two stable photoproducts, showing differentiated effects on the β‐amyloid aggregation.

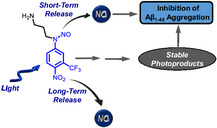

## 1 Introduction

Alzheimer's disease (AD) is a progressive neurodegenerative disorder and the leading cause of dementia worldwide, with cases expected to rise to 78 million by 2030 and 139 million by 2050 due to increasing life expectancy.^[^ ^1^ ^]^ Although AD is a multifactorial disease, a key factor in its pathogenesis is the aggregation of amyloid‐β (Aβ) peptides into toxic fibrils.^[^ ^2^ ^]^ While Aβ is synthesized as a monomer with essential functions, it can aggregate into toxic forms under pathological conditions, such as oligomers, protofibrils, and mature fibrils.^[^ ^3^ ^]^ This aggregation pathway progresses from initially benign α‐helical conformations to toxic β‐sheet structures, resulting in the formation of extracellular amyloid plaques, neurofibrillary tangles,^[^ ^4^ ^]^ cerebral amyloid angiopathy,^[^ ^5^ ^]^ and ultimately synaptic dysfunction and neuronal degeneration.^[^ ^6^ ^]^ Nitric oxide (NO) is a crucial signaling molecule involved in various physiological processes, including vasodilation, immune modulation, and neurotransmission.^[^ ^7^ ^,^ ^8^ ^]^ In AD, NO production is upregulated through the activation of inducible NO synthase (iNOS), primarily in microglial cells^[^ ^9^ ^]^ and neurons.^[^ ^10^ ^]^ The role of NO in AD is multifaceted. While traditionally associated with neuroinflammation and neurotoxicity,^[^ ^11^ ^,^ ^12^ ^]^ recent studies have highlighted its neuroprotective potential, supporting neuroplasticity, synaptic integrity,^[^ ^13^ ^]^ and long‐term potentiation.^[^ ^14^ ^]^ NO also influences Aβ aggregation through nitroso‐tyrosination of the 10th amino acid (Y10) of the protein.^[^ ^15^ ^]^ This post‐translational modification (PTM) results in the formation of 3‐nitrosotyrosine, which can undergo two‐electron oxidation, catalyzed by transition metals or other oxidants, such as H_2_O_2_, which is spontaneously generated during the early stages of the Aβ aggregation process,^[^ ^16^ ^]^ resulting in the formation of 3‐nitrotyrosine.^[^ ^17^ ^,^ ^18^ ^]^

The impact of NO‐driven PTMs on protein aggregation is debated, as some studies link them to misfolding and impairment,^[^ ^19^, ^20^, ^21^ ^]^ whereas others suggest they stabilize soluble conformations, preventing aggregation and neurotoxicity.^[^ ^22^, ^23^, ^24^, ^25^ ^]^ Such a dichotomy is not surprising. It is in line with the well‐known dependence of the NO's biological effects on its concentration^[^ ^26^ ^,^ ^27^ ^]^ and, due to its ephemeral nature, also on the generation site. This scenario makes NO‐releasing precursors bearing a high spatiotemporal control very interesting for exploiting NO's therapeutic potentials and gaining useful mechanistic insights. Light‐activatable NO precursors, namely NO photodonors (NOPDs), are ideal. The peculiar features of ease of manipulating in terms of energy, intensity, location and duration, combined with the fast rate of the photochemical reactions, make light a powerful OFF–ON trigger for introducing NO in a bioenvironment with precise control in both space and time,^[^ ^28^, ^29^, ^30^, ^31^, ^32^, ^33^, ^34^, ^35^, ^36^ ^]^ without affecting the physiological values of temperature, pH, and ionic strength.

**Scheme 1:**
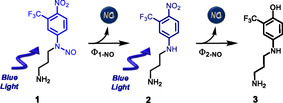
Molecular structures of **1**, **2**, and **3** and stepwise mechanism for the NO photorelease.

Stimulated by our long‐lasting interest in developing NOPDs^[^ ^28^ ^,^ ^37^, ^38^, ^39^ ^]^ for biological applications, including AD,^[^ ^40^ ^]^ in this article, we decided to investigate the effects of the multiple NOPD **1** (**Scheme** **1**) on Aβ aggregation. Compound **1** has been recently developed in our group,^[^ ^41^ ^]^ bears the very same chromophoric motif of a hydrophobic analog,^[^ ^42^ ^]^ but, in contrast, it possesses a shorter alkyl chain with an amino termination which confers excellent solubility in water. This compound was appropriately chosen because it releases two molecules of NO through a stepwise mechanism, with different rates and different quantum yields (*Φ*_NO_), under excitation with visible blue light (Scheme 1).^[^ ^41^ ^]^

These features offer the advantage of exploring the short‐ and long‐term effects of NO release on the amyloidogenic aggregation pathways. An important point to be highlighted is that NOPD **1** and its stable photoproducts **2** and **3** are characterized by the presence of the –CF_3_ moiety. Organofluorine compounds are extensively studied in biomedicine due to the unique properties conferred by fluorine.^[^ ^43^ ^]^ About 25% of all approved drugs, ranging from steroidal and nonsteroidal anti‐inflammatory, anticancer, antivirals, antibiotics and central nervous system drugs, contain at least one fluorine atom.^[^ ^44^ ^]^ Studies on Aβ adsorption onto poly(tetrafluoroethylene) surfaces have shown that fluorine promotes nontoxic α‐helix formation,^[^ ^45^ ^]^ and similar effects were observed for CF_3_‐containing solvents.^[^ ^46^ ^]^ Fluorinated analogs of diflunisal have been identified as inhibitors of transthyretin (TTR) aggregation, highlighting their potential in therapeutic interventions targeting protein aggregation diseases.^[^ ^47^ ^]^

On these bases, we investigate herein the effects of **1** and its stable photoproducts **2** and **3** on the Aβ_1–40_ aggregation under different experimental conditions. Dynamic simulation studies are also reported to shed light on the binding of all these compounds with key Aβ_1–40_ residues. We have chosen this protein as a model peptide for this mechanistic study due to its slower and more reproducible aggregation kinetics than Aβ_1–42_.

## 2 Results

**Figure 1:**
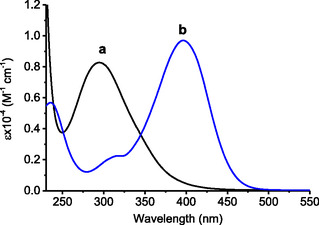
Absorption spectra of a) **1** and b) **2** in the presence of Aβ_1–40._ [Aβ_1–40_] = 10 μM; PBS (10 mM, pH 7.4): DMSO 98:2 v/v; *T* = 25 °C.

Compound **1** is based on the 4‐nitro‐3‐(trifluoromethyl)aniline scaffold, an NOPD developed in our group,^[^ ^48^ ^]^ tailored with a nitroso functionality and a short amino‐terminated alkyl chain to ensure solubility in water medium, an essential feature for biological applications. **Figure** **1** shows the absorption spectra of **1** and the non‐nitrosated analog **2** in a PBS:DMSO 98:2 v/v mixture in the presence of Aβ_1–40_. Compound **1** exhibits a main absorption band with a maximum at ≈290 nm. In contrast, the absorption of **2** is redshifted by more than 100 nm due to the push–pull character arising from the absence of the nitroso group.^[^ ^48^ ^]^ These spectral features are very similar to those observed in the same solvent in the absence of Aβ_1–40_ (data not shown), suggesting that potential interactions of both compounds with the protein (see below) do not significantly alter the chromophores’ electronic features.

**Figure 2:**
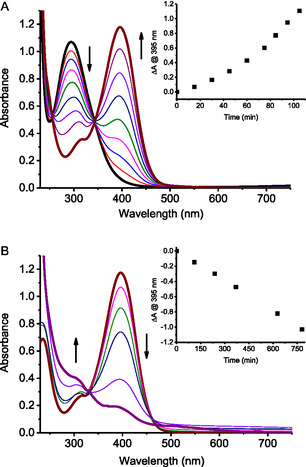
Absorption spectral changes observed upon irradiation of an air‐equilibrated solution of **1** in the presence of Aβ_1–40_ at *λ*_exc_ 405 nm at different times: A) from 0 to 105 min; B) from 105 min to 780 min. The insets show the absorbance changes at 395 nm. The arrows indicate the course of the spectral profile with illumination time. [**1**] = 125 μM; [Aβ_1–40_] = 10 μM; PBS (10 mM, pH 7.4):DMSO 98:2 v/v; *T* = 25 °C.

Irradiation of the NOPD **1** in the presence of Aβ_1–40_ with 405 nm excitation light leads to the spectral changes shown in **Figure** **2**A. They show bleaching of the 290 nm band and the formation of a new absorption at 395 nm, accompanied by clear isosbestic points indicative of a clean photochemical transformation. Prolonged irradiation leads to a bleaching of the 395 nm band accompanied by the formation of a weaker absorption below 300 nm (Figure 2B). This biphasic photochemical profile is virtually the same to that exhibited by the same compound in a different solvent in the absence of protein,^[^ ^41^ ^]^ and as well as for the hydrophobic analog of **1** retaining the same chromophoric motif.^[^ ^42^ ^]^ Accordingly, the first step involves the conversion of **1** into **2** after the homolytic rupture of the N—NO bond, typical for N‐nitrosoaniline moieties,^[^ ^49^, ^50^, ^51^ ^]^ loss of the first NO molecule, and subsequent H transfer to the anilinyl radical intermediate. The second photolytic step, typical for the 4‐nitro‐3‐(trifluoromethyl)aniline‐based chromophore of **2**,^[^ ^48^ ^]^ involves nitro‐to‐nitrite rearrangement, followed by loss of the second molecule of NO and formation of the phenol derivative **3**, after H transfer to the phenoxy radical intermediate. The quantum yields for the two sequential processes were calculated as *Φ*_1‐NO_ = 3.6 × 10^−3^ and *Φ*_2‐NO_ = 0.7 × 10^−4^, respectively. These different values allow the almost complete conversion of **1** into **2** before this latter starts its photoconversion into **3**.

**Figure 3:**
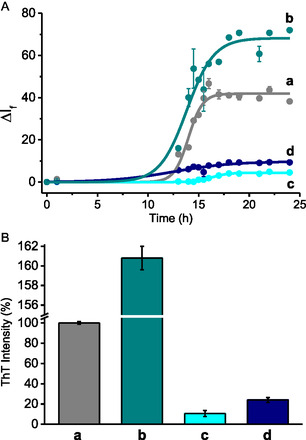
A) Fluorescence intensity changes of ThT observed in the presence of Aβ_1–40_ incubated a) alone, with **1** b) in the dark, with **1** under c) irradiation for 105 min, with **1** d) preirradiated for 105 min. B) Percentage of ThT maximum fluorescence intensity, with the a) control sample normalized to 100% and for the other samples expressed as relative percentages. Bars represent the mean ± SEM from three independent experiments, each performed with *n* = 3. [ThT] = 20 µM; [Aβ_1–40_] = 10 µM; [**1**] = 125 µM; *λ*_exc_ = 440 nm; *λ*_em_ = 482 nm; PBS (10 mM, pH 7.4):DMSO 98:2 v/v; *T* = 25 °C.

The effects of **1** on Aβ_1–40_ aggregation were assessed through Thioflavin (ThT) fluorescence assays. ThT specifically binds to *β*‐sheet structures in amyloid fibrils, causing distinct spectral changes that do not occur with either precursor polypeptides, monomers, or amorphous aggregates. Its fluorescence intensity increases proportionally to the amount of aggregated protein, enabling quantitative monitoring of Aβ_1–40_ fibrils.^[^ ^52^ ^]^ When the protein monomers were incubated with compound **1** in the dark, ThT fluorescence intensity increased by up to 160% (**b** in **Figure** **3**A,B) compared to the untreated control (**a** in Figure 3A,B) suggesting a proaggregative effect of **1** under these experimental conditions. In contrast, irradiation of **1** in the presence of Aβ_1–40_ for 105 min (approximately end of the first photolytic step) led to a remarkable reduction of the ThT fluorescence, reaching ≈10% of the control level (**c** in Figure 3A,B). Based on the molar absorptivity of **1** and **2** (Figure 1) and the spectral changes observed in Figure 2A, it can be estimated that such an irradiation time yields ≈117 µM of **2**, ≈8 µM **3**, and ≈125 µM of NO.

The potential effect of NO on the tyrosine residue was investigated by recording its fluorescence spectrum during Aβ_1–40_ incubation with **1** (Figure S1, Supporting Information). Incubation in the dark for 105 min left the typical tyrosine fluorescence with maximum at ≈305 nm almost unchanged. In contrast, irradiation of the mixture for the same interval time under otherwise identical experimental conditions led to pronounced fluorescence quenching.

To selectively assess the impact of the stable photoproducts **2** and **3** on the observed antiaggregative effect, an additional experiment was performed in which **1** was preirradiated for 105 min before adding Aβ_1–40_. In this way, the irradiated mixture contains the same amount of **2** and **3** as above (no differences in the *Φ*_NO_ values were observed in the absence of protein) and, in addition, the nitrite ions arising from the relatively rapid oxidation of NO under aerobic conditions.^[^ ^53^ ^,^ ^54^ ^]^ Under these conditions, ThT fluorescence dropped to ≈20% of the control (**d** in Figure 3A,B). To disclose if this relevant inhibitory effect is due to the produced nitrite, the stable organic photoproducts, or both, a further experiment was carried out by incubating the protein with nitrites alone at a concentration of 125 µM. No relevant change in ThT fluorescence intensity was observed compared to the untreated sample (Figure S2, Supporting Information), ruling out the participation of this species in the antiaggregation effect.

**Figure 4:**
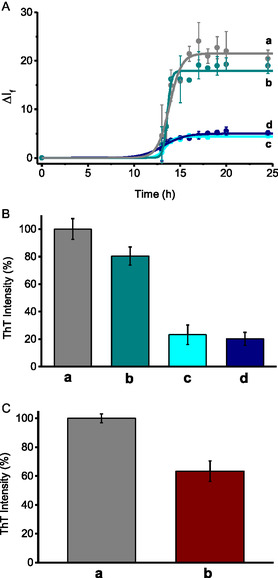
A) Fluorescence intensity changes of ThT observed in the presence of a) Aβ_1–40_ incubated alone, with b) **2** in the dark, with c) **2** under irradiation for 780 min, with d) **2** preirradiated for 780 min. B) Percentage of ThT fluorescence intensity, with the control sample a) normalized to 100% and for the other samples expressed as relative percentages. C) Percentage of ThT maximum fluorescence intensity, with the control sample normalized to a) 100% and for A*β*_1–40_ incubated with **3** b) expressed as relative percentages. Bars represent the mean ± SEM from three independent experiments, each performed with *n* = 3. PBS (10 mM, pH 7.4): DMSO 98:2 v/v; *T* = 25 °C. [ThT] = 20 µM; [A*β*_1–40_] = 10 µM; [**2**] = 117 µM; [**3**] = 8 µM; *λ*_exc_ = 440 nm; *λ*_em_ = 482 nm; PBS (10 mM, pH 7.4):DMSO 98:2 v/v; *T* = 25 °C.

The role of **2** and **3** in the protein aggregation was therefore thoroughly explored through ad hoc designed experiments. Specifically, nonirradiated **2** was tested at a concentration equivalent to that generated from the photolysis of **1** after 105 min (≈117 µM), yielding ThT fluorescence intensity only to 80% of the untreated sample (**b** in **Figure** **4**A,B).

Then, **2** was irradiated in the presence of Aβ_1–40_ for ≈780 min (corresponding almost to the end of the second photolytic step). In this case ThT fluorescence further decreased to ≈20% (**c** in Figure 4A,B). However, when **2** was preirradiated for the same time and only after incubated with Aβ_1–40_, the same decrease in the fluorescence intensity as in the sample irradiated in the presence of Aβ_1–40_ was observed (**d** in Figure 4A,B).

Further investigations were performed using **3** as model compound at a concentration of ≈8 µM, corresponding to the estimated amount formed from **1** after the first photolysis step (Figure 2A). These experiments reduced the ThT fluorescence to ≈60% of the control value (**d** in Figure 4C). It is important to emphasize that the results related to all the experiments with ThT are not trivial because: 1) although the NOPDs absorb in the same spectral region of ThT, the fluorescence emission of this latter was always corrected for its actual fraction of absorbed photons; 2) in the experiments under light irradiation, ThT was always added rigorously after the irradiation steps, ruling out any artifact arising from potential photodegradation of the probe; and 3) all compounds investigated were stable under the ThT assay conditions by their unaltered absorption spectra recorded at the beginning and at the end of the kinetic experiments.

**Figure 5:**
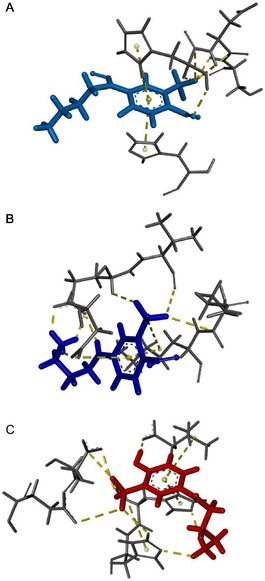
3D representations of the orientations adopted by A) **1**, B) **2,** and C) **3** when submitted to MD simulations with Aβ_1–40_ monomer.

In silico molecular dynamics (MD) simulations were finally performed to gain insights into the potential binding of all compounds investigated with the protein. **Figure** **5**A–C shows the final 3D conformations of **1**, **2**, and **3**, respectively. The interaction maps and the interaction legend are reported in Figure S3, Supporting Information. **Table** **1** reports the interactions of the three compounds with the key amino acid residue of Aβ_1–40_, considered crucial for the aggregation process. For each residue, the Table reports its physicochemical classification and the type of interaction established. **1** interacts with Aβ_1–40_ through a hydrophobic contact with His14. **2** engages Lys16 through its CF_3_ group, while also forming halogen bonds with Val12 and Gln15. The terminal NH_2_ group of **3** forms a H‐bond with His13, which also participates in a π–π interaction with the phenolic ring. His14 further contributes by interacting with the NH_2_ group and establishing both π–alkyl contacts and hydrogen bonds with the CF_3_ moiety. The OH group of **3** also forms a H‐bond with Val40.

**Table 1:** Properties of the interactions of **1**, **2**, and **3** with key amino acid residues involved in Aβ_1–40_ aggregation.

Molecule	Residue	Type of amino acid	Type of interaction
**1**	His14	Polar	T‐shaped *π*‐*π*
**2**	Lys16 Val12 Gln15	Charged Hydrophobic Polar	*π*‐alkyl Halogen bond Halogen bond
**3**	His13 His14 Val40	Polar Polar Hydrophobic	H‐bond *π*‐*π* stacked H‐bond C—H/*π* bond *π*‐alkyl H‐bond *π*‐alkyl

## 3 Discussion

**Scheme 2:**
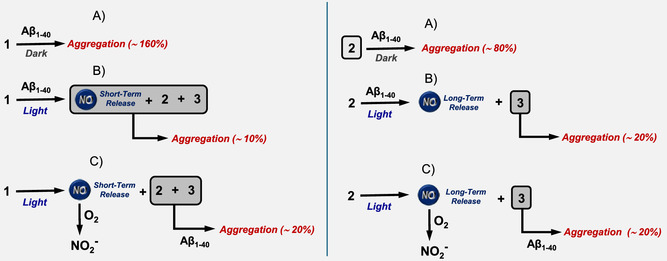
Schematic representation of the effects of **1** and its photoproducts **2** and **3**, under different experimental conditions, on the Aβ_1‐40_ aggregation percentage (in brackets) revealed by ThT assay. The species participating in the inhibitory effect are highlighted in the box. A,B) Conditions in which compounds **1** and **2** are incubated with Aβ_1‐40_ and kept in the dark or irradiated with blue light for 105 min and 780 min, respectively. C) Compound **1** and **2** preirradiated for 105 and 780 min, respectively, and then incubated with Aβ_1‐40_.

Irradiation of the NOPD **1** in the presence of Aβ_1–40_ leads to releasing two molecules of NO through a stepwise mechanism (Figure 2A,B), leading to **2** and **3** as stable photoproducts according to Scheme 1. The sequential NO release occurs in two remarkably different time windows with the first and second processes completed in ≈105 min and 780 min, respectively. This feature allows investigation of the effects of the same concentration of NO produced in the early and late stage of the aggregation process of Aβ_1–40_, as well as of the two stable photoproducts generated. A general overview of the results obtained is illustrated in **Scheme** **2** and discussed in the following.

NOPD **1,** incubated with Aβ_1–40_ in the dark, significantly encourages protein aggregation (A in Scheme 2, left). However, when a similar sample is exposed to blue light irradiation for 105 min, a remarkable inhibitory effect on the aggregation of the protein is observed (B in Scheme 2, left). Under these experimental conditions, the irradiation generates 117 µM of **2**, ≈8 µM **3**, and ≈125 µM of NO. The experiments performed with **1** preirradiated for the same time and then incubated with the protein allow gaining insights into the actual species contributing to this strong antiaggregative effect (C in Scheme 2, left). Under these conditions, NO is converted into nitrite by oxidation with molecular oxygen. However, experiments performed with nitrite did not show any influence on the aggregation kinetics. In contrast, the two stable photoproducts **2** and **3** incubated with Aβ_1–40_ reduced its aggregation, although to a lesser extent than observed upon direct irradiation of **1**. This finding provides clear‐cut evidence for the direct participation of NO generated during the early stage of the aggregation kinetic, in the antiaggregative effect. A possible mechanism might involve the nitrosation of the tyrosine residue, followed by its oxidation to 3‐nitrotyrosine. This process is likely facilitated by H_2_O_2_ spontaneously generated during the initial phases of amyloid aggregation.^[^ ^15^ ^,^ ^16^ ^,^ ^18^ ^]^ This hypothesis is supported by the pronounced quenching of the typical tyrosine fluorescence observed exclusively after irradiating **1** in the presence of Aβ_1–40_ for 105 min and absent in the dark (Figure S1, Supporting Information). This finding is in line with the occurrence of photogenerated NO‐driven PTMs yielding low fluorescent nitro‐ and nitrosotyrosine derivatives.

The individual contributions of **2** and **3** and NO generated in the late stage of the aggregation kinetics were evaluated using a second set of experiments. Incubation of **2** with Aβ_1–40_ in the dark had only a little antiaggregative action (A in Scheme 2, right). On the other hand, when the same compound was irradiated in situ for 780 min (almost the end of the second photolytic process), the aggregation was significantly reduced (B in Scheme 2, right). Under these experimental conditions, the irradiation generates ≈125 µM of **3** and a similar amount of NO. Interestingly, when **2** was preirradiated for the same time and then incubated with Aβ_1–40_ in the dark, the same extent of antiaggregative effect was observed (C in Scheme 2, right). Since this sample contains nitrite, which has no effect on the aggregation kinetics (see above), and ≈125 µM of **3**, the very similar outcome revealed in the case B and C (Scheme 2 right) rules out any participation of NO in the antiaggregative effect which is almost exclusively due to compound **3**. This is an important point to be highlighted. It demonstrates that despite NO being produced at similar concentration generated by the direct irradiation of **1**, it is less effective when released over a longer time window, during more advanced phases of the aggregation process. Under these conditions, the oxidation of 3‐nitrosotyrosine to 3‐nitrotyrosine at Y10 is likely impaired due to the absence of the early stages released H_2_O_2_.^[^ ^15^ ^,^ ^16^ ^,^ ^18^ ^]^

The results obtained with individuals **2** and **3** clearly show a higher antiaggregative properties of the latter (A and C in Scheme 2, right). Therefore, the strong reduction observed in the preirradiation experiments with **1** (C in Scheme 2. left) where the concentration of **2** (117 µM) is much higher than **3** (8 µM) suggests a synergistic action of **2** and **3** in inhibiting the protein aggregation. Such behavior aligns well with the observations reported by Török et al., who demonstrated that CF_3_‐substituted indole derivatives exhibit strong antiamyloid activity in vitro.^[^ ^55^ ^]^ Their study highlighted the crucial role of the CF_3_ group in enhancing the acidity of the hydroxyl group, thereby promoting peptide binding and fibrillogenesis inhibition. Notably, the absence of the CF_3_ or OH group completely abolished the inhibitory effect.^[^ ^56^ ^]^

The in silico studies revealed that all the investigated compounds interact with key amino acid residues involved in amyloid aggregation, namely polar,^[^ ^57^ ^,^ ^58^ ^]^ charged,^[^ ^59^ ^,^ ^60^ ^]^ hydrophobic,^[^ ^51^, ^52^, ^53^, ^54^, ^55^, ^56^, ^57^, ^58^, ^59^, ^60^, ^61^, ^62^, ^63^ and terminal residues.^[^ ^64^ ^,^ ^65^ ^]^ Compound **1**, identified as the only proaggregative species in the ThT assays, engages in a single interaction, limited to its aromatic ring. In contrast, compound **2**, which showed a moderate antiaggregative effect, establishes a more extended network of contacts with key aggregation‐prone residues. Finally, the phenol derivative **3**, which exhibited excellent aggregation inhibitory properties, forms an even broader interaction network involving at least six distinct types of interactions with critical amino acid residues. All the residues mentioned in the previous results section are known to play a pivotal role in the amyloidogenic aggregation process.^[^ ^66^ ^]^ Notably, many of the observed interactions are directly or indirectly mediated by the CF_3_ group. In addition, its strong electron‐withdrawing effect likely increases the acidity of the adjacent OH moiety, enhancing its ability to engage in H‐bonding with surrounding residues. These findings highlight the unique capacity of organofluorinated compounds to modulate Aβ_1–40_ aggregation by tuning specific intermolecular interactions.

## 4 Conclusions

In summary, our findings highlight that the proaggregative properties of **1** turn into excellent antiaggregative activities upon light exposure and stepwise conversion into its photoproducts, **2** and **3** after sequential NO photorelease. This approach further enabled assessing the impact of NO release on the formation of mature Aβ_1–40_ fibrils. When NO is generated in the early stage of the protein aggregation, it reduces it, likely by promoting Y10 nitrosation to 3‐nitrosotyrosine. This may further be oxidized into 3‐nitrotyrosine, possibly driven by early‐stage aggregation byproducts such as H_2_O_2_. Conversely, a similar amount of NO produced over a longer time window failed to exert the same antiaggregative effect. The phototriggered NO release enabled precise control over its timing, location, and dosage, preventing excessive oxidative conditions and offering valuable insights into its role in amyloid aggregation under controlled conditions that replicate the NO levels associated with iNOS upregulation during inflammation.^[^ ^67^ ^]^

Stable photoproducts **2** and **3** generated after releasing the first and second molecules of NO also have antiaggregative properties, with **3** being much more effective than **2**. In both cases, the CF_3_ moiety was found to play a key role in interacting with crucial amyloidogenic residues. Notably, in the case of compound **3** the CF_3_ moiety enhanced the acidity of the OH group, promoting hydrogen bonding with Aβ_1–40_ and further expanding its interaction network with the protein. These findings underscore the potential of organofluorinated compounds in modulating amyloid aggregation through specific physicochemical effects. Note that, although this study has focused on Aβ_1–40_, which is less pathogenic and exhibits different aggregation behavior than Aβ_1–42_, both peptides share the same 40‐residue core. This suggests that the insights from Aβ_1–40_ on stage‐dependent NO effects and organofluorinated photoproducts interactions might be useful for future investigations on Aβ_1–42_, which is highly associated with AD. Studies to better assess how these PTMs and photoproduct molecules influence preformed small Aβ_1–40_ oligomers or even physiological monomeric species are underway in our laboratories.

## 5 Experimental Section

5.1

5.1.1

##### Chemicals

All chemicals were purchased by Sigma–Aldrich and used as received. Amyloid β peptide 1–40 (Aβ_1–40_) (purity > 95%) was obtained from GenScript. Organic solvents were removed under reduced pressure at 30 °C. Synthetic‐purity solvents were used. All solvents used for the spectrophotometric studies were spectrophotometric grade. Ultrapure water (MilliQ) was used. Compounds **1** and **2** were prepared as previously reported.^[^ ^41^ ^,^ ^68^ ^]^

##### Samples preparation

All solutions were prepared using PBS (10 mM, pH 7.4): DMSO (98:2) as a solvent. The concentrations of **1** and **2** were determined spectrophotometrically, using *ε*_290nm_ = 8,500 M^−1^ cm^−1^ and *ε*_400nm_ = 9,600 M^−1^ cm^−1^, respectively. Solutions of compound **3** at the desired concentration were obtained by irradiating a H_2_O:MeOH 1:1 v/v solution of **2**, at a known concentration until complete photoconversion was achieved, ensuring that only **3** remained as the sole stable photoproduct.^[^ ^48^ ^]^ The solution was then evaporated to dryness and redissolved in PBS. Aβ_1–40_ was initially dissolved in HFIP at 1 mg mL^−1^, and the solvent was evaporated to obtain a monomeric film, which was subsequently redissolved in DMSO to a final concentration of 0.5 mM. A 10 µM Aβ_1–40_ solution was then prepared by diluting the monomeric stock in the previously prepared PBS solutions, ensuring a consistent DMSO content of 2% across all samples. The resulting solutions were either maintained in the dark or exposed to blue light (*λ*_exc_ = 405 nm) using a continuous laser in a thermostated quartz cuvette (*T* = 25 °C, pathlength = 1 cm, capacity = 3 mL). Blue light exposure was carried out before or after adding Aβ_1–40_ monomers, depending on the irradiation protocol employed (preirradiation or in situ irradiation, respectively).

##### NO Photorelease Quantum Yields

Photodecomposition quantum yields for **1** (*Φ*_1_‐NO) and **2** (*Φ*_2_‐NO) were determined at *λ*_exc_ = 405 nm, within the 20% transformation range, using the following equation

*Φ*_NO_ = [C]xV / t x(1–10^−A^)xI

where [C] corresponds to the concentration of the phototransformed **1** or **2**, V is the irradiated sample volume, t is the irradiation time, A is the sample absorbance at the excitation wavelength, and I represents the intensity of the light source. The amounts of phototransformed **1** and **2** were determined spectrophotometrically by monitoring the absorption changes at 395 nm, using *Δε* at 395 = 10.000 M^−1^· cm^−1^. The excitation light intensity was evaluated by potassium ferrioxalate actinometry.

##### ThT Assays

To evaluate the impact of all compounds on Aβ_1–40_ amyloid aggregation, we performed the ThT assay following established protocols.^[^ ^69^ ^]^ Briefly, all preprepared samples containing Aβ_1–40_ (10 μM) and the test compounds at the required concentration were incubated with ThT (20 μM) at 25 °C for 25 h. ThT was added after irradiation, when required by the specific experimental setup, to prevent photodegradation, using a 15 mM PBS stock solution diluted to 20 µM. Its concentration was determined spectrophotometrically, using *ε*_412nm_ = 36,000 M^−1^ cm^−1^.^[^ ^70^ ^]^All the fluorometric measurements were carried out in triplicate at *λ*_exc_ = 440 nm and *λ*_em_ = 482 nm. The collected fluorimetric data were fitted to the following equation.

(1) $$F\left(t\right)=\frac{F_{\max}-F_{0}}{1+e^{-\frac{t-t_{1/2}}{k}}}$$

F_max_ − *F*_0_ represents the maximum fluorescence increment during amyloid‐type aggregation. This value was reported for all samples as a percentage relative to that measured in the seed‐free Aβ_1–40_ sample.

The lag phase (*t*_lag_), defined as the time period before the formation of amyloid species, can be calculated using the following equation

(2) $$t_{lag}=t_{1/2}-2/k$$

All the kinetics were followed for 25 h and were conducted with freshly prepared samples.

##### Instrumentation

UV–vis absorption and fluorescence emission spectra were recorded using a Jasco V‐560 spectrophotometer and a Spex Fluorolog‐2 (model F‐111) spectrofluorometer, respectively, in air‐equilibrated solutions with 1 cm path length quartz cells. Irradiations were performed in a thermostated quartz cell (1 cm path length, 3 mL capacity) under gentle stirring, using a continuum laser with *λ*_exc_ = 405 nm with a beam diameter of 1.5 mm and an irradiance on the samples of ≈175 mW cm^−2^.

##### Statistical Analysis

All ThT assays were performed in triplicate (*n* = 3). Data were normalized to the Aβ + ThT control (*F*_max_ = 100%) and expressed as percentages relative to the control. Results were presented as mean ± standard deviation. Statistical significance was assessed by one‐way analysis of variance followed by Tukey's post‐hoc test, and all treated samples were significantly different from the control (*p* < 0.05). Data processing and analyses were performed using OriginPro (OriginLab Corporation).

##### In Silico Analysis

Marvin Sketch was used to create the 2D chemical structures, and the same software's MMFF94 force field was used to apply molecular mechanics energy minimization to each structure.^[^ ^71^ ^]^ The 3D geometry of all compounds was then optimized using PM3 Hamiltonian,^[^ ^72^ ^]^ as implemented in the MOPAC 2016 package, assuming a pH of 7.4.

MD simulations were performed using GROMACS‐4.5.3,^[^ ^73^ ^]^ employing PDB: 1aml for Aβ_1–40_ monomer.^[^ ^74^ ^]^ All simulations were performed in the isothermal–isobaric ensemble in a periodic simulation cell 60 × 40 × 40 Å. The pressure was controlled at 1 atm, and the temperature was retained at 310 K using the Parrinelo–Rahman barostat and V‐rescale thermostat.^[^ ^75^, ^76^, ^77^ ^]^ A two‐femtoseconds (fs) time step was used to integrate the equation of motion. Electrostatic interaction was calculated using particle mesh Ewald sums with a nonbonded cut off 10 Å.^[^ ^78^ ^]^ Bonds between hydrogen and heavy atoms were constrained at their equilibrium length using the linear constraint solver algorithm.^[^ ^79^ ^]^ Initially, the energy minimizations of the system were carried out followed by the equilibration of all the systems for 200 picoseconds (ps). Later, production runs of 50 ns were performed in all the systems. The 0.15 M NaCl was added to mimic the physiological conditions. The trajectories were saved at 10 ps intervals for further analysis. BIOVIA Discovery Studio Visualizer was used to unravel interactions between Aβ_1–40_ monomer and molecules.

## Conflict of Interest

The authors declare no conflict of interest.

## Supporting information

Supplementary Material

## Data Availability

The data that support the findings of this study are available on request from the corresponding author. The data are not publicly available due to privacy or ethical restrictions.
