# Supplementary material for: Multifaceted Effects of a Multiple Nitric Oxide Photoreleaser and its Photoproducts on Amyloid‐β Aggregation
Source: Chembiochem. 2025 Nov 24;27(1):e202500667. doi: 10.1002/cbic.202500667 (PMC12781159; doi:10.1002/cbic.202500667)
Supplement: Supplementary file 1 — Supplementary Material [file CBIC-27-e202500667-s001.pdf]

Supporting Information

Multifaceted Effects of a Multiple Nitric Oxide Photoreleaser and its Photoproducts on Amyloid-β Aggregation

Francesca Laneri, Cristina Parisi and Salvatore Sortino*

PhotoChemLab, Department of Drug and Health Sciences, University of Catania, I-95125, Catania, Italy;

*Corresponding author: ssortino@unict.it

- **Figure S1……………………………………………………………………………2**
- **Figure S2……………………………………………………………………………3**
- **Figure S3……………………………………………………………………………4**

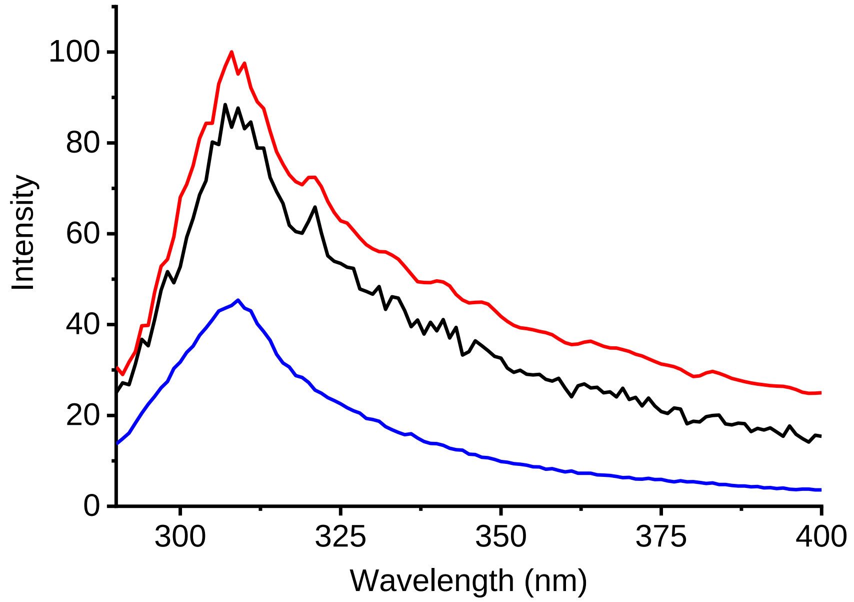

Figure S1. Fluorescence emission spectra (λ_{exc}= 280 nm) of A$\beta_{1\text{-}40}$ in the presence of **1** after 0 (black) and 150 min of incubation in the dark (red), and after 150 min of irradiation (blue). The spectra were corrected for both the fraction of light absorbed by A$\beta_{1\text{-}40}$ at the excitation wavelength and the fraction of fluorescence re-absorbed by **1** (before irradiation) or **2** (generated after photolysis of **1**). [A$\beta_{1\text{-}40}$] = 10 μM; [**1**] = 125 μM; PBS (10 mM, pH 7.4):DMSO = 98:2 v/v; T = 25 °C.

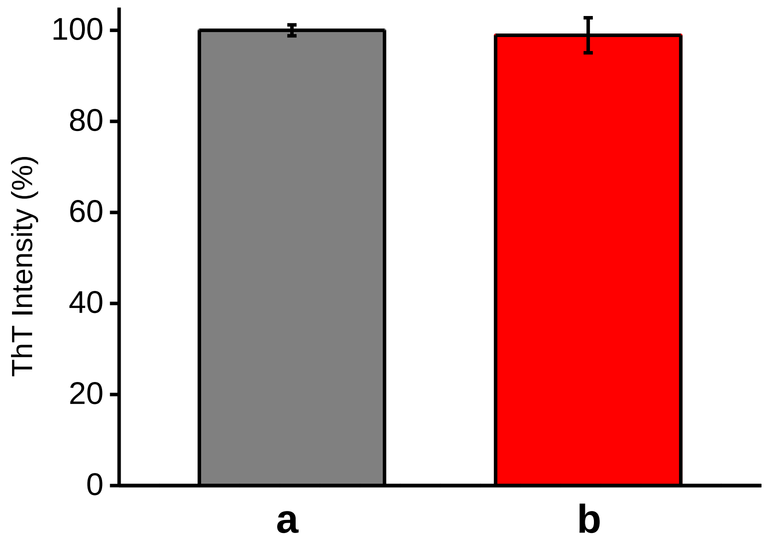

Figure S2. Percentage of ThT maximum fluorescence intensity, with the control sample normalized to 100% (**a**) and for Aβ_{1-40} incubated with nitrites expressed as relative percentages (**b**). Bars represent the mean ± SEM from three independent experiments, each performed with n = 3. PBS (10 mM, pH 7.4): DMSO 98:2 v/v; T = 25 °C. [ThT] = 20 μM; [Aβ1-40] = 10 μM; [NO_2^-] = 125 μM; PBS (10 mM, pH 7.4):DMSO 98:2 v/v; T = 25 °C.

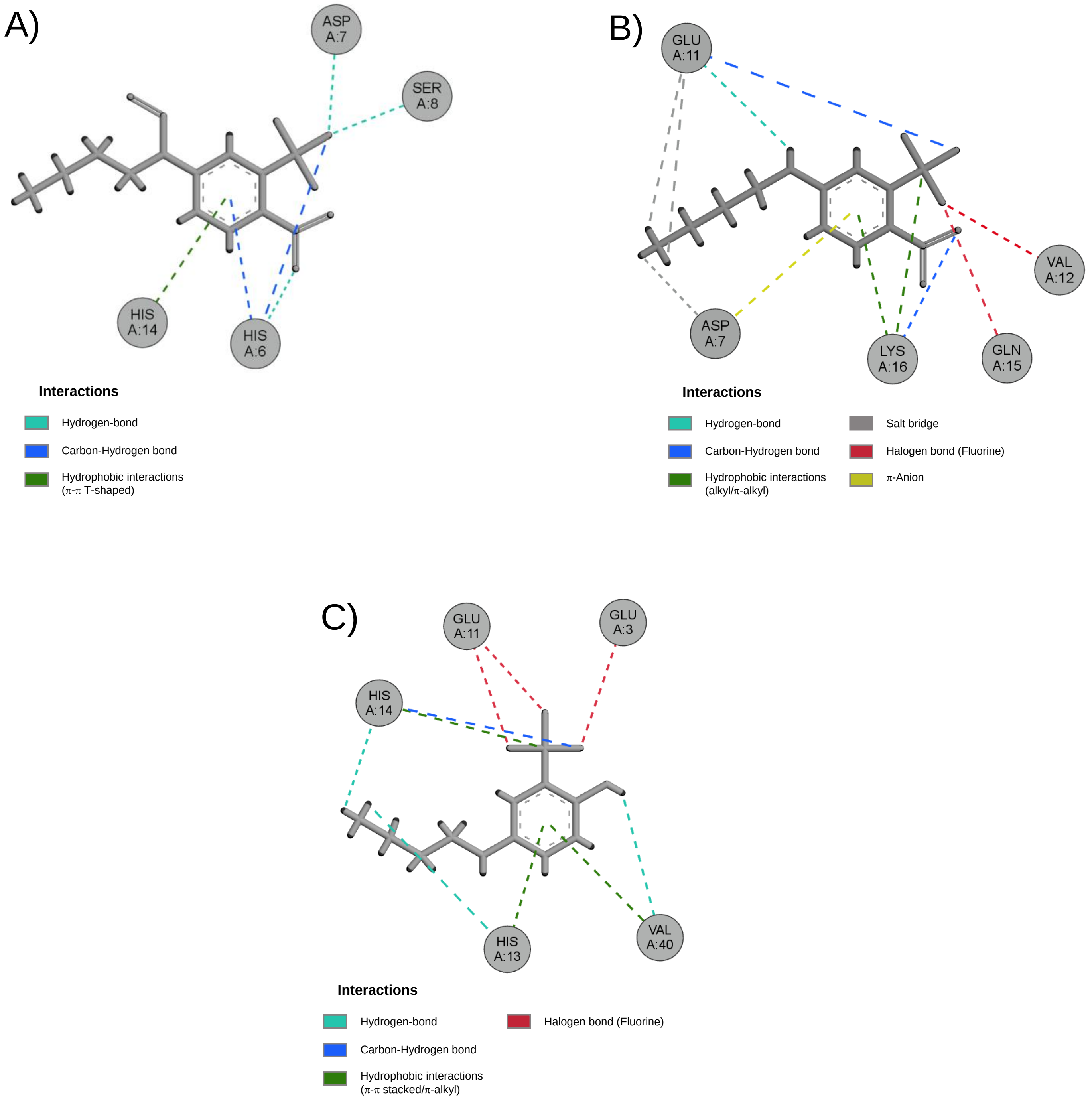

Figure S3. 2D representations of the orientations adopted by (A) **1**, (B) **2** and (C) **3** when submitted to MD simulations with Aβ_{1-40} monomer.
